# Primary Biliary Cholangitis and Bile Acid Farnesoid X Receptor Agonists

**DOI:** 10.3390/diseases8020020

**Published:** 2020-06-10

**Authors:** Ludovico Abenavoli, Anna Caterina Procopio, Sharmila Fagoonee, Rinaldo Pellicano, Marco Carbone, Francesco Luzza, Pietro Invernizzi

**Affiliations:** 1 Department of Health Sciences, University “Magna Graecia”, 88100 Catanzaro, Italy; procopioannacaterina@gmail.com (A.C.P.); luzza@unicz.it (F.L.); 2 Institute of Biostructures and Bioimaging (CNR) c/o Molecular Biotechnology Center, 10126 Turin, Italy; sharmila.fagoonee@unito.it; 3 Unit of Gastroenterology, Molinette Hospital, 10126 Turin, Italy; rinaldo_pellican@hotmail.com; 4 Division of Gastroenterology, Center for Autoimmune Liver Diseases, Department of Medicine and Surgery, University of Milano-Bicocca; 20126 Milan, Italy; marco.carbone@unimib.it (M.C.); pietro.invernizzi@unimib.it (P.I.); 5 European Reference Network on Hepatological Diseases (ERN RARE-LIVER), San Gerardo Hospital, 20900 Monza, Italy

**Keywords:** liver, ursodeoxycholicacid, obeticholic acid, chenodeoxycholic acid, scaffold

## Abstract

Primary biliary cholangitis (PBC) is a chronic autoimmune liver disease characterized by the progressive destruction of the intrahepatic bile ducts. Currently, the first line drug for PBC is ursodeoxycholic acid (UDCA) characterized by anti-apoptotic, anti-inflammatory and protective actions on cholangiocytes. Despite its recognized therapeutic action, 30–40% of PBC patients only partially benefit from UDCA therapy. This has led to the identification of the role of the farnesoid x receptor (FXR) in cholestatic liver diseases and, consequently, to the development of obeticholic acid (OCA), a steroid FXR agonist that has been recently approved for the treatment of PBC. OCA though is not effective in all patients and can cause itch, which eventually induces treatment drop out. Therefore, the search for new therapeutic strategies for PBC has begun. This review, in addition to summarizing the current treatments for PBC, provides overview of the chemical characteristics of new steroid FXR agonist candidates that could represent a future perspective for the treatment of PBC.

## 1. Introduction

Primary biliary cholangitis (PBC) is a chronic autoimmune liver disease characterized by the progressive destruction of the intrahepatic bile ducts and progression, if not treated, to fibrosis and cirrhosis [1,2,3]. PBC predominantly affects women over the age of 40 (woman/man rate of 9–10:1). Recent epidemiological data point to an increase in cases in men (woman/man rate of 2–3:1) [4]. The disease is characterized by a yearly incidence and prevalence rate of 0.33–5.8/100.000 and 1.91–40.2/100.000 individuals, respectively [4,5,6]. Most of the affected subjects are asymptomatic throughout the initial phase of the disease, which is often diagnosed by chance following an increase in alkaline phosphatase (ALP). Diagnosis is made when at least two of the following criteria are met: (1) positivity for antimitochondrial antibodies (AMA), (2) increase in ALP, (3) chronic non-suppurative cholangitis of small and medium caliber bile ducts [7,8].

Although the etiopathogenesis of PBC is still uncertain, multifactoriality represents a crucial element in the onset of the pathology. The development of PBC has been linked to genetic predisposition of the affected subjects. In particular, PBC susceptibility has been shown to be associated with polymorphisms in the Human Leucocyte Antigen (HLA) region especially in the alleles DRB1*08, DRB1*11, DRB1*14, DPB1*03:01 and DQB1 [9,10,11,12].

One of the determining factors is the loss of tolerance for the PDC-E2 autoantigen (component E2 of pyruvate dehydrogenase complex). It seems that PDC-E2 plays a fundamental role in activating Th1 cells response through interleukin (IL)-12. T cells start producing interferon (INF)-γ and tumor necrosis factor (TNF)-α, which trigger a cytotoxic action. In addition, IL-4 activates B cells causing the release of AMA, which promote senescence and apoptosis of bile epithelial cells (BEC) [13,14,15]. In addition, another element contributing to the onset of PBC is the presence of a defective “bicarbonate umbrella”. This “bicarbonate umbrella” is essential for protection against the accumulation of bile acids and their cytotoxic effects. In PBC, the impairment of this protective “umbrella” increases the sensitivity of cholangiocytes to bile acids, which accumulate in the BEC generating reactive oxygen species thus further promoting senescence and apoptosis [14].

Those observations have prompted research efforts to identify compounds that could promote the elimination of bile acids regulating their synthesis, absorption and excretion. In this context, particularly important are the farnesoid X receptor (FXR) agonists, such as OCA, which has recently been approved for the treatment of PBC as a second-line drug [14]. Currently, several other therapeutic agents are undergoing evaluation in order to identify new pharmacologically active compounds.

The purpose of this review is to offer an overview of the compounds showing promising activities in PBC, and above all, it aims to highlight the chemical characteristics of new steroid FXR agonists in order to clarify their structure-activity relationship.

## 2. The First-and Second-Line Therapies for PBC

The first-line pharmacological treatment in PBC patients is ursodeoxycholic acid (UDCA), and in those with inadequate response to UDCA treatment, the FXR agonist OCA, which represents the second-line treatment for PBC, is used. Despite the therapeutic efficacy of UDCA and OCA, interesting pharmacological alternatives are being evaluated as reported below.

### 2.1. UDCA

UDCA represents the first line therapy in the treatment of PBC, and for a long time, it has been the only drug approved by the Food and Drug Administration (FDA) for this syndrome [16]. Therapy with UDCA involves long-term treatment with a dose of 13–15 mg/kg/day [17]. UDCA can be administered as a single oral dose, and in case of poor tolerability, the dose can be divided [14]. UDCA owes its effectiveness in the treatment of PBC, to a series of effects such as the protective action on cholangiocytes, anti-apoptotic and anti-inflammatory activities and a post-translational stimulation of synthesis of liver export pumps [1,2]. Indeed, it has been shown that one of the protective mechanisms of UDCA against cholestasis is the disposal of accumulated bile acids. However, the secretory capacity of the hepatocytes is closely related to the presence of transport proteins in the canalicular membrane. In this regard, UDCA induces an increase in expression for bile salt export pumps (BSEP), proteins associated with multi-drug resistance (MDR3) and multidrug resistance associated protein 4 (MRP4) thus facilitating the elimination of bile acids [18,19,20]. Furthermore, timely diagnosis and early treatment with UDCA delay the progression of PBC. In particular, it was found that treatment with UDCA of PBC patients at advanced stages (extensive liver fibrosis) led to a considerably reduced rate of disease progression. Moreover, after 4 years of therapy, an arrest in the initial state of the syndrome has been observed in 76% of cases. Despite the high efficacy demonstrated by UDCA therapy, about 40% of the subjects do not benefit from its use [2]. In this case, the combined regimen, UDCA plus OCA, has been proposed.

### 2.2. OCA

OCA is a semisynthetic derivative of chenodeoxycholic acid (CDCA) and represents a second-line therapy in case of non-responsiveness to UDCA [1,16,17]. OCA is an FXR agonist with 100 times higher affinity for the receptor with respect to CDCA, the endogenous ligand [16]. FXR is a member of the nuclear receptor superfamily, comprising endocrine, metabolic and orphan receptors [21]. FXR is highly expressed in the liver, gallbladder, intestines and kidneys [22,23]. In particular, upon activation, FXR regulates the synthesis, excretion, transport, absorption and metabolism of bile acids [24]. Furthermore, OCA increases the expression of fibroblast growth factor 19 (FGF-19) thus leading to a reduction in the synthesis of biliary acids. In the ileum, OCA reduces the reabsorption of biliary acids by down-regulating the apical transporter (apical sodium dependent bile acid transporter or ABST) [16]. OCA, unlike UDCA that interacts at a post-transcriptional level, acts directly on the synthesis, absorption and secretion of bile acids [14].The efficacy of OCA was assessed in the POISE study, a double-blind phase 3 study of 12-month duration [25]. In this study, 217 patients with inadequate response to UDCA were divided into three groups and were treated with OCA, respectively, with a daily dose of 10 mg, 5 mg with an adjustment to 10 mg when applicable, or with placebo in addition to UDCA treatment (13–15mg/kg/day). The primary end point, represented by an ALP level of less than 1.67 × upper limit normal, occurred in 46% of patients in 5–10 mg OCA treatment and in 47% of those in 10 mg OCA treatment compared to 10% in the placebo group. In addition, improvements in cholestasis, hepatocellular damage, inflammation and apoptosis were observed. The most commonly observed side effect was pruritus, noted in 56% of the 5–10 mg group and 68% of the 10mg group versus 38% of the placebo group. Currently, in the clinical setting, OCA is used in combination with UDCA in subjects who do not respond to UDCA treatment alone; this combination allows to reduce the incidence of liver complications [2]. OCA is considered as a safe treatment, albeit itching is the more common side effect [26].

## 3. New Compounds for the Treatment of PBC

**Figure 1:**
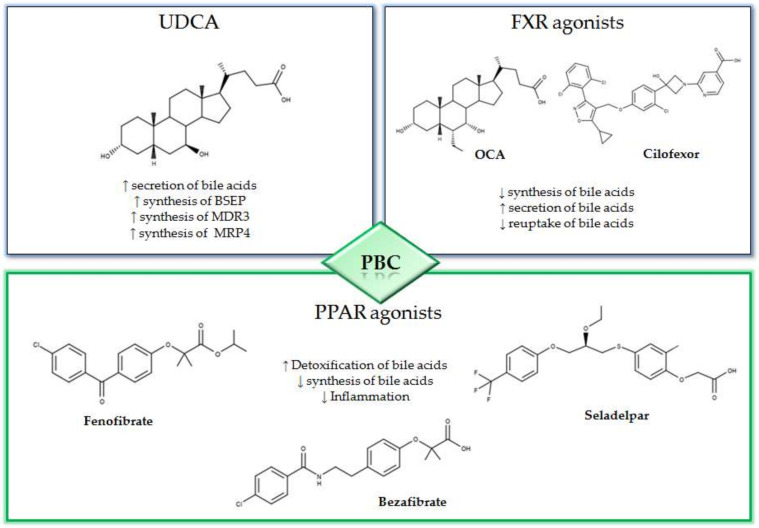
Mechanisms and 2D structures of Ursodeoxycholic acid (UDCA), Obeticholic acid (OCA), Cilofexor, Fenofibrate, Bezafibrate and Seladelpar.

The peroxisome proliferator-activated receptor agonists (PPAR) have shown promising therapeutic activity in PBC. In fact, the activation of PPAR is associated to an anti-cholestatic action and a reduction in the synthesis of bile acids with a decrease in the liver inflammation (Figure 1) [27,28]. Among the most promising PPAR activators that are currently being evaluated for the treatment of PBC, there are bezafibrate, fenofibrate and seladelpar. In the BEZURSO study (NCT01654731), a phase 3 double blind study of 24 months duration evaluated the efficacy of bezafibrate [29]. In this study, 100 patients with an inadequate response to UDCA, according to the Paris 2 criteria, were enrolled. The patients were randomized into two groups; one group was treated with 400 mg of bezafibrate and UDCA and the other with placebo and UDCA. The primary outcome was defined by a complete biochemical response with normal levels of total bilirubin, ALP, aminotransferase, albumin and a normal prothrombin index. The primary outcome occurred in 31% of patients assigned to bezafibrate/UDCA treatment and in 0% of those in the placebo/UDCA group. In addition, normal levels of ALP were observed in 67% of patients treated with bezafibrate/UDCA and in only 2% of those in the placebo/UDCA group [29]. In a recent study, 118 patients previously treated with UDCA for at least 1 year were enrolled and treated with combined bezafibrate/UDCA therapy for 1 year [30]. The GLOBE and UK-PBC scores generated with UDCA monotherapy were compared with those obtained after 1 year of bezafibrate/UDCA therapy. The mean GLOBE score determined by UDCA therapy alone was 0.504 ± 0.080 while, after one year of bezafibrate/UDCA combination therapy, this value was 0.115 ± 0.085. Furthermore, the addition of bezafibrate did not ameliorate the real rates of liver transplantation or liver-related death with respect to the predicted UK-PBC risk score before the addition of bezafibrate, while the predicted risk was significantly reduced by addition of bezafibrate (*p* < 0.0001) [30].

Fenofibrate has also been evaluated for its effects in patients with CBP. In particular, a retrospective study was conducted on 120 non-responder patients to UDCA [31]. Patients were divided into two groups: one group was treated with fenofibrate and UDCA (FF group), the other group was treated with UDCA only (UDCA group). Significant improvements in the biochemical parameters were observed in the FF group, in particular the final average ALP in the FF and UDCA groups was 184 ± 98 and 274 ± 172 IU/L, respectively (*p* = 0.002). Furthermore, the fenofibrate-treated group had a significantly lower level of alanine aminotransferase (ALT) (78 ± 47 IU/L versus 43 ± 34 IU/L, *p* = 0.001) and aspartate aminotransferase (AST) (60 ± 25 IU/L versus 44 ± IU/L, *p* = 0.05) at baseline [31]. Moreover, fenofibrate was evaluated in a retrospective cohort study consisting of 23 PBC patients with an inadequate response to UDCA monotherapy [32]. All patients were treated with fenofibrate and UDCA combination. With respect to the baseline values, a significant decrease in median serum ALP levels after 3 months (*p* = 0.0002), 6 months (*p* < 0.0001) and 12 months (*p* < 0.0001) of treatment with fenofibrate/UDCA was observed. The side effects more frequently reported were nausea, vomiting and abdominal pain, but the therapy was stopped in six patients for intolerance or fenofibrate-induced liver injury [32].

Seladelpar a selective PPAR-δ agonist, previously referred to as MBX-8025, has also shown promise in the treatment of PBC. In particular, in a double-blind phase 2 study of 12-week duration (NCT02609048) the efficacy of seladelpar was evaluated in patients with an inadequate response to UDCA [33]. Patients were divided into three groups and were treated respectively with 50 mg or 200 mg of seladelpar or with placebo. The results indicated that patients treated with seladelpar 50 mg or 200 mg presented an ALP reduction, compared to baseline, of 53% and 63%, respectively, while in the placebo group the reduction was only 2% [33]. Despite this promising activity, during the study, a grade 3 increase in ALT was observed among side effects, and for this reason, the study had been interrupted.

### 3.1. NGM282

The NGM282 (aldafermin) is an engineered analogue of FGF19, a growth factor which is produced following the activation of FXR and acts in the liver by regulating the synthesis of bile acids. In a double-blind study of 28-day duration (NCT02026401), the efficacy of NGM282 was assessed in 45 PBC patients with inadequate UDCA response [34]. Patients were randomized into three groups and were treated with 0.3 mg, 3 mg of NGM282 or placebo, respectively. The reported results indicated that at the end of the treatment, ALP levels decreased at both 0.3 mg (least squares (LS) mean –51.0 IU/L (standard error (SE) 15.4)) and 3 mg (–66.0 IU/L (SE 16.0)) NGM282 compared to placebo (3.3 IU/L (SE 14.8)). Furthermore, significant changes were observed in γ-glutamyl transferase (GGT) values and C4 levels, the metabolite produced during the synthesis of 7-α-hydroxycholesterol [34].

### 3.2. Non-Bile Acids FXR Agonists

Cilofexor, previously referred to as (GS-9674), is a non-bile acid FXR agonist. In the double-blind phase II study (NCT02943460), the effects of cilofexor (30 mg, 100 mg or placebo orally for 12 weeks) were evaluated in 52 PSC patients [35]. Promising results in terms of reduction in biochemical markers of cholestasis were observed in the study. In particular, a significant decrease in serum ALP (median reduction −21%; *p* = 0.029 versus placebo) and GGT (−30%; *p* < 0.001) was observed after 12 weeks of treatment with 100 mg of cilofexor. Furthermore, in both groups treated with cilofexor, there was a reduction in the metabolite produced by the conversion of cholesterol to 7-α-hydroxolesterol by CYP7A1, and it was only after treatment with 100 mg of cilofexor that there was a reduction in total bile acids, primary and secondary bile acids compared to placebo [35]. Tropifexor is a recently identified FXR agonist that has demonstrated promising steatohepatitis reduction activity [36,37]. The safety of tropifexor administration was assessed in a study on healthy volunteers, and the results indicated that the drug is well tolerated with an acceptable safety profile [38]. A clinical trial on the efficacy of tropifexor in patients with non-alcoholic steatohepatitis is nearly completed (NCT02855164).

### 3.3. Immunomodulatory Strategies

PBC is an autoimmune disease that affects the bile ducts; nevertheless, the immunomodulatory therapies have not been well characterized. In this regard, ustekinumab, a monoclonal antibody directed against interleukin-12 and inteleukina-23, has been evaluated in a phase two study on PBC patients [39]. The subjects enrolled in the study, characterized by an inadequate response to UDCA, received subcutaneous treatment with ustekinumab, but no therapeutic activity was observed. In addition, abatacept, a fusion protein formed by the Fc region of immunoglobulin IgG1 molten into the extracellular domain of cytotoxic T-lymphocyte antigen 4 (CTLA-4), was tested in a 24-week study on PBC patients with inadequate response to UDCA [40]. This study indicated that abatacept is inefficacious for this pathology since almost all patients did not reach the primary endpoint. In fact, the study showed that the treatment did not induce changes in ALT, albumin, bilirubin and immunoglobulin levels.

## 4. Structure–Activity Relationship of Bile Acid FXR Agonists

Since in PBC a protective effect conferred by the activation of FXR has been observed, several attempts were made to identify compounds with great selectivity for FXR without or with few side effects. The design and synthesis of new FXR agonists are based on the use, as the starting scaffold, of an endogenous bile acid, the CDCA. Several modifications in the structure of CDCA were made in order to devise highly specific compounds.

**Figure 2:**
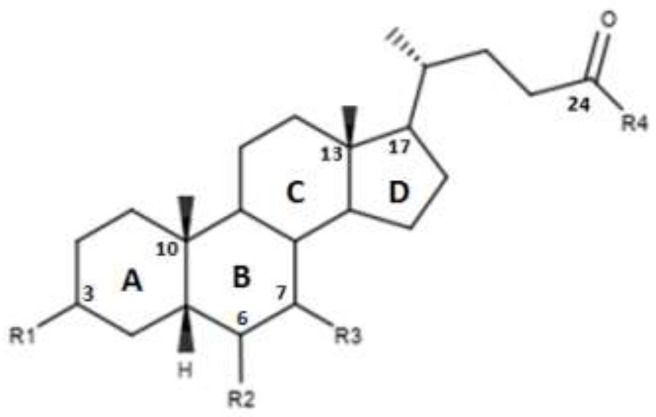
2D image of the changes to bile acids scaffold reported in Table 1.

CDCA is a molecule, resulting from the fusion of four rings denominated A, B, C and D. The main scaffold of CDCA, which consists of a cyclopentanofenanthrenic unit, is characterized by the presence of two α OH in C- 3 and C-7, two β methyl in C-10 / C-13 and from a side chain on carbon C-17. This structure plays a fundamental role in the chemical-pharmacological activity of the molecule and was the starting point for the design of new, more powerful and selective FXR agonists. Some changes made to the CDCA scaffold led to the identification of pharmacological agents currently used in therapy such as OCA and promising new bile FXR agonists (Figure 2, Table 1). Specifically, it has been observed that the introduction of an alkyl group on the C-6 of ring B significantly improved the activity [41]. In particular, in OCA, and in compounds 1, 2 and 3, ethyl, methyl, propyl and butyl groups were introduced respectively in C-6. The optimal activity was observed for OCA with an ethyl group in C-6. On the contrary, the insertion of a butyl moiety in C-6 as in compound 3 resulted in the formation of an inactive compound [41]. Thus, it was shown that the introduction of a small alkyl function in C-6 can improve the functionality of the compounds and assisted in designing compounds like OCA. A study carried out by Festa et al. evaluated the activity of FXR agonists following modifications on C-3 and C-7 [42]. It was observed that the elimination of α-OH in C-3 in compound 4 did not lead to activity loss for FXR, indicating that the presence of α-OH in C-3 was not essential for interaction at the receptor level. Moreover, compound 5 (Table 1), which lacked α-OH in C-3 and bore a -NHCH_2_CH_2_SO_3_Na group in position C-24, was devoid of FXR activity [42]. Compounds 6 and 7, which had no α OH in C-3 but were endowed with β OH in C-7 were also inactive on FXR. This suggests that the presence of the α-OH group in C-7, unlike the α-OH in C-3, is fundamental for the agonism towards FXR. Compound 8 characterized by an epimerization of OH in C-3 and bearing α-OH in C-7 had higher capacity than CDCA in activating the FXR [42].

Pellicciari et al. evaluated the effects of further modifications on the C-6 with the introduction of alkyl chains and polar groups as shown in Table 1 [43]. The compounds 9 and 11, characterized respectively by an allyl and 2-propionyl in C-6, demonstrated a discrete activity towards FXR, while the compound 10 characterized by a 2-hydroxyethyl in C-6 demonstrated a low activity towards FXR. This is consistent with the observation that the presence of an alkyl chain in C-6 improves the affinity for FXR. On the contrary, compounds 12 and 13 with α-OH and α-OCH_3_ in C-6 presented reduced activity [43]. The importance of an apolar group in C-6 is thus important. Furthermore, the compounds 14 and 15, characterized by α/β fluorine in C-6, exhibited an intermediate behavior due to the peculiarities of the hydrophilic–lipophilic character of the fluorine [43].

**Figure 3:**
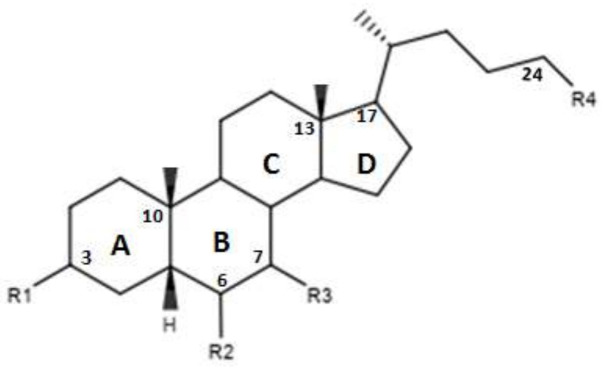
2D image of the changes to bile acids scaffold reported in Table 2.

In Figure 3 and in Table 2 were reported the characteristics of a series compounds in which the C-24 carboxylic function has been modified. In particular, Festa et al. observed that compound 16 maintained the FXR agonist activity despite the elimination of the OH group in C-3 and the reduction of the carboxyl function in C-24 [42]. Compound 17, devoid of OH in C-3 and characterized by a -OSO_3_Na group in C-24, showed a low activity for the FXR [42]. Thus, the replacement of the carboxylic function in C-24 with -OSO_3_Na may be crucial in generating powerful FXR agonists. Unlike compounds 16 and 17, compound 18, which was devoid of OH in C-3 and endowed with -OSO_3_Na in C-7 and C-24 did not demonstrate activities for FXR [42]. Compound 19, which had β-OH in C-7, was inactive, unlike compound 16, which represented its epimer in C-7. This evidence once again underlines the importance of the α-OH group in C-7, which is necessary to maintain the agonist activity for FXR. In support of these observations, compounds 20 and 21, characterized by a β-OH in C-7, showed no activity. Compound 22 devoid of OH in both C-3 and C-7 confirmed the poor activity on FXR [42]. On the contrary, compound 23, despite the absence of OH in C-3 and C-7, showed good activity on FXR. However, on the whole, the elimination of OH group in C-7 proved to be harmful to the activity of the agonists [42]. D’Amore et al. proposed a series of compounds with -OSO_3_Na on C-24 and substitutions on C-6 carbon [44]. More specifically, the compounds 24, 25, 26 and 27 (Table 2), with α-OH in C-3 and C-7 and an -OSO_3_Na group in C-24, were evaluated following the addition of an ethyl chain in C -6. This study showed that compound 25 with an ethyl chain on C-6 had an activity comparable to that of CDCA. In particular, INT-767, which represents the sodium salt of compound 25, has been employed in a completed phase 1 clinical study to evaluate its safety and pharmacokinetics in healthy subjects. On the other hand, the compound 24 without a chain on C-6 did not have significant activities. Therefore, this confirms the importance of the presence of an ethyl chain in the C-6 position. Moreover, compounds 26 and 27 characterized by -OSO_3_Na in C-3, C-7 and C-24 did not demonstrate significant activity on FXR [44]. Compounds 28, 29, 30 and 31 without the C-6 alkyl chain and characterized by a β-OH/-OSO_3_Na group in C-7 have shown little activity on FXR. These evidences confirm the importance of the alkylic chain in C-6 and of α-OH group in C-7 [44].

**Figure 4:**
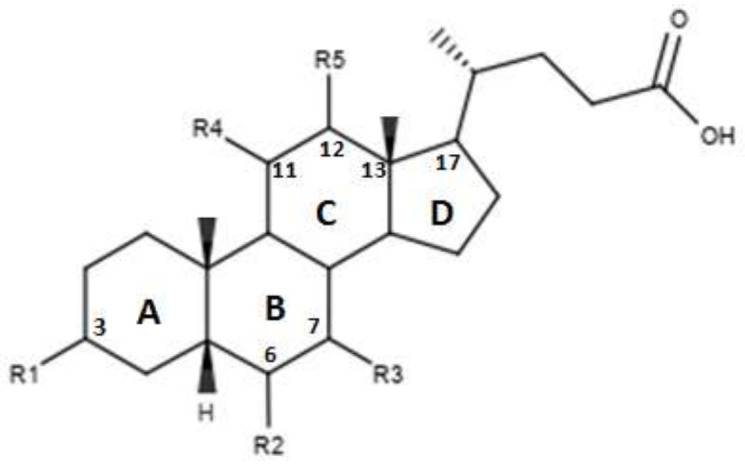
2D image of the changes to bile acids scaffold reported in Table 3.

Pellicciari et al proposed a series of derivates, the characteristics of which are shown in Figure 4 and in Table 3. [45]. These molecules are characterized by the introduction of a hydroxyl group in C-11 for compounds TC-100 and 32, and in C-12 for compounds 33 and 34 [45]. The results reported by this study indicate that these derivatives have a good agonistic activity on FXR. Of note, in the case of compound TC-100, the introduction of a β OH C-11 group, led to the formation of a compound with high activity and selectivity towards FXR characterized by an EC_50_ = 0.14µM [45]. Thus, the introduction of polar functions in the C ring is important and can contribute to the creation of bonds at the receptor site level [45].

**Figure 5:**
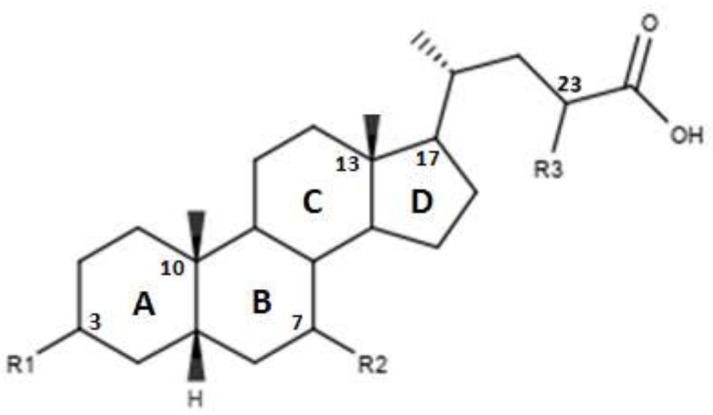
2D image of the changes to bile acids scaffold reported in Table 4.

Pellicciari et al. proposed two derivatives in which a OH was inserted on C-23, the characteristics of which are shown in Figure 5 and in Table 4 [43]. The functionalization of C-23, seen in the compounds 35 and 36, was found to reduce or abolish the agonist activity. These results suggest that replacing C-23 with polar or apolar groups reduce the affinity for FXR [43].

**Figure 6:**
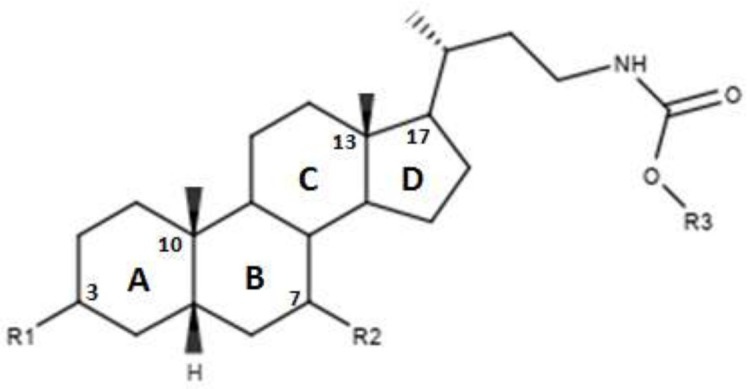
2D image of the changes to bile acids scaffold reported in Table 5.

The Authors also proposed a series of modifications in CDCA resulting in the formation of carbamate derivatives, the characteristics of which are shown in Figure 6 and in Table 5 [46]. As indicated in Table 5, these carbamate derivatives were functionalized through alkyl groups, cycloalkyl and aromatic rings. From the cell-based luciferase assay carried out, it was shown that the compound 42 presented a higher activity than CDCA on FXR. In particular, compound 42 had the highest score as FXR agonist [46]. Compounds 37, 38, 39, 40 and 41 showed partial activity on FXR. Thus, the functionalization of the side chain with the formation of carbamate derivatives may lead to particularly effective compounds and warrants further analysis [46].

## 5. Conclusions

Our review provides a summary of the therapies currently used in PBC patients and summarizes the efforts made in finding compounds with promising agonistic activities on FXR. Different changes made to the CDCA scaffold in order to identify improved FXR agonists useful in PBC are reported. In particular, the importance of the α-OH group in C-7 and an alkyl group in position C-6 is highlighted. Furthermore, a series of carbamate and sulfonate derivatives, which present interesting perspectives for new pharmacologically active compounds as well as the most interesting chemical characteristics of the steroid FXR agonists, are reported herein.

## Figures and Tables

**Table 1:** Modifications made on bile acids scaffold.

Compounds	R_1_	R_2_	R_3_	R_4_	EC_50_ (µM)
OCA	α-OH	α-ethyl	α-OH	OH	0.099 ± 0.01
1	α-OH	α-methyl	α-OH	OH	0.75 ± 0.08
2	α-OH	α-propyl	α-OH	OH	1.11 ± 0.13
3	α-OH	α-butane	α-OH	OH	>30
4	H	H	α-OH	OH	-
5	H	H	α-OH	NHCH_2_CH_2_SO_3_Na	-
6	H	H	β-OH	OH	-
7	H	H	β-OH	NHCH_2_CH_2_SO_3_Na	-
8	β-OH	H	α-OH	OH	-
9	α-OH	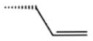	α-OH	OH	0.48
10	α-OH	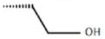	α-OH	OH	61.15
11	α-OH	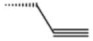	α-OH	OH	0.54
12	α-OH	α-OH	α-OH	OH	>30
13	α-OH	α-OCH_3_	α-OH	OH	14.73
14	α-OH	α-F	α-OH	OH	15.11
15	α-OH	β-F	α-OH	OH	1.21

**Table 2:** Modifications made on bile acids scaffold.

Compounds	R_1_	R_2_	R_3_	R_4_	Ec50 (µM)
16	H	H	α-OH	OH	-
17	H	H	α-OH	OSO_3_Na	~9
18	H	H	α OSO_3_Na	OSO_3_Na	-
19	H	H	β-OH	OH	-
20	H	H	β-OH	OSO_3_Na	-
21	H	H	β-OSO_3_Na	OSO_3_Na	-
22	H	H	H	OH	-
23	H	H	H	OSO_3_Na	-
24	α-OH	H	α-OH	OSO_3_Na	-
25	α-OH	α-ethyl	α-OH	OSO_3_Na	~1
26	α-OSO_3_Na	H	α-OSO_3_Na	OSO_3_Na	-
27	α-OSO_3_Na	α-ethyl	α-OSO_3_Na	OSO_3_Na	-
28	α-OH	H	β-OH	OSO_3_Na	-
29	α-OSO_3_Na	H	β-OSO_3_Na	OSO_3_Na	-
30	α-OSO_3_Na	H	β-OH	OSO_3_Na	-
31	α-OSO_3_Na	H	β-OH	OH	-

**Table 3:** Modifications made on bile acids scaffold.

Compounds	R_1_	R_2_	R_3_	R_4_	R_5_	EC_50_ (µM)
TC-100	α-OH	α ethyl	α-OH	β-OH	H	0.14 ± 0.05
32	α-OH	α ethyl	α-OH	α-OH	H	3 ± 2
33	α-OH	α ethyl	α-OH	H	β-OH	4.7 ± 3.0
34	α-OH	α ethyl	α-OH	H	α-OH	7.0 ± 2.3

**Table 4:** Modifications made on bile acids scaffold.

Compounds	R_1_	R_2_	R_3_	EC_50_ (µM)
35	α-OH	α-OH	β-OH	13.20
36	α-OH	α-OH	α-OH	10.23

**Table 5:** Modifications made on bile acids scaffold.

Compounds	R_1_	R_2_	R_3_	EC_50_ (µM)
37	α OH	α OH	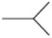	2.50
38	α OH	α OH	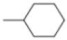	1.14
39	α OH	α OH	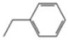	0.79
40	α OH	α OH	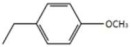	1.48
41	α OH	α OH	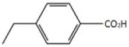	7.11
42	α OH	α OH	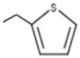	0.41
